# Long-term follow-up of HIV-infected patients in salvage therapy with raltegravir plus optimized background regimens: a multicentre Italian experience

**DOI:** 10.1186/1758-2652-13-S4-P38

**Published:** 2010-11-08

**Authors:** S Landonio, P Meraviglia, AF Capetti, A Di Biagio, S Lo Caputo, G Sterrantino, A Ammassari, B Menzaghi, M Franzetti, G De Socio, G Pellicanò, E Mazzotta, P Zucchi, G Rizzardini

**Affiliations:** 1 AO Luigi Sacco, I° Division of Infectious Diseases, Milan, Italy; 2 AO Luigi Sacco, II° Division of Infectious Diseases, Milan, Italy; 3 San Martino Hospital, Infectious Diseases Clinic, Genoa, Italy; 4 Santa Maria Annunziata Hospital, Infectious Diseases Clinic, Florence, Italy; 5 Careggi Hospital, Infectious Diseases Clinic, Florence, Italy; 6 Spallanzani Hospital, III Division of Infectious Diseases, Rome, Italy; 7 AO Busto Arsizio, Infectious Diseases Clinic, Busto Arsizio (Varese), Italy; 8 AO Luigi Sacco, Infectious Diseases Clinic, Milan, Italy; 9 Santa Maria della Misericordia Hospital, Infectious Diseases Clinic, Perugia, Italy; 10 Policlinico G. Martino Hospital, Infectious Diseases Clinic, Messina, Italy; 11 Pescara Hospital, Infectious Diseases Clinic, Pescara, Italy; 12 I Division Infectious Diseases, AO Luigi Sacco, Milan, Italy

## Background

Raltegravir is the first integrase inhibitor to get into the clinic and the MK-0518 Expanded Access Program (EAP) started in Italy in 2007, so that most patients now have an observation period of two years. Up to date a few studies have described the durability of raltegravir-containing regimen with a follow up of 96 weeks [1].

## Methods

Patients from the MK0518 EAP were followed-up prospectively since enrolment in the study. Clinical and laboratory data were collected every 2 - 4 months after commercial availability.

## Results

Out of 250 patients enrolled in the MK0518 EAP in our centres, 229 are still on the same regimen. The mean follow up was 80 weeks. At the time of present analysis (96 weeks) 133 patients were evaluated. Eighty-nine% of these had HIV-RNA < 50 Cp/mmc. Two patients temporarily stopped therapy, one developing the integrase 72I mutation plus 2 additional RT mutations, and both stably resuppressed the virus with the same regimen. Seven patients discontinued therapy due to virological failure: primary mutations for integrases were detected in all the samples (155H/N, 148H+140S, 143R) in addition to other mutations (157Q, 72I, 73V, 165I, 97A, 163R). Four patients with non-primary mutations detected continued the same therapy maintaining viral replication below 4000 copies/ml, with 1.9 log decrease in HIV-RNA. One patient was lost to follow up, four discontinued due gastrointestinal AEs and one for CK elevation, while eight died during the observation period, 4 of non-Hodgkin’s lymphoma (NHL), 1 of acute myocardial infarction, 2 of end stage liver disease and 1 post-transplantation for HCV-related cirrhosis. The immunological gain of the patients who remained on therapy is good in a salvage setting: +230 CD4/mmc.

## Conclusions

The MK0518 EAP seems to have contributed in a relevant measure to obtain full viral suppression for two years in most of our salvage patients, with an important immunologic gain and very few adverse events, in a situation were active companion drugs were really difficult to find out (the overall mean GSS was <2). The number of deaths (including NHL that represented the cause of death for 50% of the cases) has not been related to the drug regimen, but more likely to the advanced stage of HIV infection.
